# Thykamine Extracts from Spinach Reduce Acute Inflammation In Vivo and Downregulate Phlogogenic Functions of Human Blood Neutrophils In Vitro

**DOI:** 10.3390/biomedicines8070219

**Published:** 2020-07-16

**Authors:** Vickie Beaupré, Nathalie Boucher, Isabel Desgagné-Penix

**Affiliations:** 1 Department of Chemistry, Biochemistry and Physics, Université du Québec à Trois-Rivières, Trois-Rivières, QC G8Z 4M3, Canada; Vickie.Beaupre@uqtr.ca; 2 Pharmaceutical plant, Devonian Health Group, Montmagny, QC G5V 4T1, Canada; nboucher@groupedevonian.com; 3 Plant Biology Research Group, Trois-Rivières, QC G8Z 4M3, Canada

**Keywords:** inflammation, antioxidants, thylakoids, spinach, neutrophils, phytomolecules

## Abstract

The anti-inflammatory and antioxidant role of Thykamine, a botanical extract of thylakoides obtained from spinach leaves, has been investigated in animal and cellular models. The oxidative properties have been proven by inhibiting NO production (>98%) in J774A.1 cells and by protecting a linoelic acid emulsion subjected to lipid peroxidation caused by AAPH. Thykamine injected intraperitoneally to rats reduced the inflammatory process of (TNBS)-induced colitis and carrageenan-induced paw edema. As neutrophils are the first cells to migrate to inflammatory sites, the influence of Thykamine on the primary neutrophil functions were studied. Thykamine dose-dependent reduced neutrophil chemiotaxis, phagocytosis, and degranulation. No change in the release of LDH by neutrophils on Thykamine was recorded. Thykamine inhibited by 85% the neutrophil production of O_2_^−^. A superoxide recovery activity was observed on a zymography demonstrating a SOD-like enzyme on Thykamine extracts. Spontaneous fluorescence provided by carotenoid and chlorophyll pigments (488/675 nm) detected Thykamine on the surface, in the cytoplasm (mainly central where Golgi are present) and weakly in the nucleus of neutrophils. The results argue that SOD and pigments found in Thykamine are part of its antioxidant and anti-inflammatory properties shown in in vivo and in vitro models of inflammation.

## 1. Introduction

Medicinal plants and their extracts have been used by humans for centuries to feed and treat themselves. The antioxidant and anti-inflammatory properties of botanical extracts are among those with very high potential for the development of new drugs. Plant extracts can have anti-inflammatory and immunoregulatory properties through different active components, in particular antioxidants like flavonoids and carotenoids [1,2,3,4]. In a recent study, the antioxidant capacity of a spinach extract, constituted of enriched thylakoid membrane extracts and defined as Thykamine has been demonstrated [5]. Thylakoid membranes composing this extract are found in photosynthetic organisms. They form a network of aggregated membranes attached to unstacked membranes in which galactolipids represent the main lipid constituents. The particular organization of these membranes promotes optimal activity of the two photosystems I and II (PSI and PSII), protein complexes in which photochemical reactions take place and responsible of photosynthesis activity. The antennae complexes of these two photosystems contain carotenoid pigments conferring to thylakoid extracts such as Thykamine a high antioxidant capacity. Moreover, PSI, being rich in Fe-S clusters which are sensitive to reactive oxygen species (ROS)s, are protected by superoxide dismutase (SOD) enzymes located at the external surface of the membrane. SOD reacts with superoxide to form hydrogen peroxide, neutralizing ROS. These antioxidant properties represent powerful weapons against oxidative and nitrosative stresses occurring when there is an imbalance in the production of reactive oxygen species and/or reactive nitrogen species (RNS). Oxidative stress contributes to the development of the inflammatory and immune response of some chronic diseases leading to the cellular dysfunctions and tissue destructions. As a corollary, the different biochemical structure and enzyme activities of Thykamine could be involved in the anti-inflammatory and immunoregulatory functions of thylakoid membranes found in spinach leaves.

The therapeutic effects of Thykamine were previously reported. For example, immuno-modulatory properties of Thykamine on alveolar macrophages were demonstrated through regulation of pro- and anti-inflammatory cytokine production [6]. Moreover, it was shown that Thykamine contained various antioxidant molecules and enzymes involved in protecting and restoring the harmful effects of UV exposure [7]. Since neutrophils are the most abundant cellular subset and the first migrating cells accumulating at sites of inflammation with possible deleterious effects, we proposed that Thykamine could affect neutrophils function at these sites.

The inflammatory response to injury implicates an obligatory infiltration of leukocytes in tissue, and among leukocytes, neutrophils are the first migrating cells. Recruitment of neutrophils to sites of inflammation must involve chemotaxis, adhesion of neutrophils to endothelium, trans-endothelial diapedesis, and extravascular migration of neutrophils. From the signals of attraction, neutrophils enter an activation status including the generation of reactive oxygen intermediates and arachidonic acid metabolites, phagocytosis, and the release of proteolytic enzymes. Major chemotactic factors for neutrophils are non-specific products such as formyl-methionyl-peptides, split products of the fifth component of complement, leukotriene B_4_, and specific attractants such as chemokines (i.e., interleukin 8 (IL-8). The effector functions of neutrophils are fully manifested after their directional influx and resultant local accumulation at sites of tissue reactions. These functions are activated by phagocytosis and soluble stimuli, and depend on both the release of granular contents such as lysosomal hydrolases and the expression of membrane associated enzyme systems such as the superoxide-generating NADPH oxidase. However, besides microbicidal activities and degradation of particles, hydrolytic and oxidative enzymes from neutrophils granules may contribute to deleterious inflammation and damage of host tissues.

Thykamine, being a mixture of various components (thylakoids and galactolipids), corresponds to the “multi-target/multi-component” approach. In this study, the anti-inflammatory activities of Thykamine were investigated in two inflammatory animal models. The antioxidant properties of Thykamine, including superoxide anions (O_2_^−^) scavenging and protection against lipid peroxidation and nitric oxide (NO) production were also examined in cell models. We hypothesized that these antioxidant properties combined to the anti-inflammatory activities observed in the animal models could interfere with the inflammatory functions of human blood neutrophils. We studied whether Thykamine could modulate primary functions of freshly isolated human blood neutrophils in vitro, such as chemotaxis, phagocytosis, production of superoxide anions, and degranulation, and how Thykamine could enter the cell.

## 2. Materials and Methods

### 2.1. In Vivo Rodent Models of Inflammation

TNBS-induced colitis in rats. Colonic inflammation was induced by using the technique of Morris et al. with slight modifications [8]. Male Wistar rats (Charles River laboratories, Sherbrooke, QC, Canada) were kept in individuel cages, at 20 °C and 55% relative humidity with 12-h light/12-h dark cycle. The rat experiment was approved by the Animal Experiment committee of the Charles River Laboratories (Montreal, QC Canada) on 15 April 2002 and conducted in accordance with the Canadian Council on Animal Care and Use. The rats were subjected to an 18-h starving period prior to 5 treatments. Briefly, male Wistar rats (180–200 g) after an overnight food deprivation, were anesthetized with isoflurane before insertion of a polyethylene catheter of 8 cm into the colon via the anus. The hapten TNBS at 25 mg/mL dissolved in 50% aqueous/ethanol (vol/vol) was injected into rat colon (total volume injected: 1 mL/rat). The control rats received 1 mL of vehicle (aqueous/ethanol, vol/vol) intracolonically. Rats were injected intraperitoneally with Thykamine (50, 5, or 0.5 mg/kg) in sterile physiologic saline (1 mL) immediately prior to anesthesia. These doses were selected based on previous studies [6,7].

Carrageenan-induced paw edema in rats. Male Wistar rats (180–200 g) which had been fasted overnight (18 h) received Thykamine (0.5, 5 or 50 mg/kg) in sterile physiologic saline by intraperitoneal injection (1 mL) immediately prior to subplantar injection of carrageenan (0.1 mL of 1% suspension in 0.9% saline) in the right hind paw [9]. Paw thickness was measured from ventral to dorsal surfaces, with a dial caliper. Paw circumference was measured immediately prior to carrageenan injection and 5 h afterwards. Edema was expressed in mm as the increase of paw circumference measured after carrageenan injection and compared to the pre-injection value for individual animals.

### 2.2. Reagents

Dextran T-500 and Ficoll-Paque were obtained from Pharmacia Biotech (Montréal, QC, Canada). fMLP, human LF, anti-human LF antibody and O-dianiside hydrochloride were obtained from Sigma Chemical Co (St-Louis, MO, USA). Chemotaxis plates (96 wells) were purchased from Neuroprobes, Gaithersburg, MD, USA. LTB4 was from Cascade Biochem Ltd. (Reading, Berkshire, England). IL-8 was from Peprotech Canada Inc. (Ottawa, ON, Canada). Fluorescein-labeled *Escherichia coli* K-12 BioParticles, calcein-AM and cytochrome *c* (125 mg/mL) were from Molecular Probes, Invitrogen Canada Inc. (Burlington, ON, Canada). Anti-CD63 Fluorescein isothiocyanate (FITC)-labeled and anti-CD66b PE-labeled antibodies were from Beckman Coulter, Inc. (Mississauga, ON, Canada). Thylakoid extracts (Thykamine) were provided by Devonian Health Group (Montmagny, QC, Canada). Thykamine extract were solubilized at 100 mg/mL (5%) in phosphate buffered saline (PBS).

### 2.3. Neutrophil Preparations

Neutrophils were obtained from venous blood of healthy as previously described [10]. Contaminating erythrocytes were eliminated by a hypotonic lysis (15 s, RT). After 2 washes, neutrophils were resuspended in Hank’s Balanced Salt solution (HBSS) containing 10 mM HEPES pH 7.4, 1.6 mM Ca^++^, and no magnesium. Differential cell counts of leukocytes were performed by flowcytometry (EPICS-XL, Beckman Coulter), Wright’s and non-specific esterase stains. Final neutrophil suspensions were more than 98% pure with no CD3 positive cells and non-specific esterase positive cells represented less than 0.1% of the cell population. Viability was greater than 98% as routinely assessed by trypan blue dye exclusion. When appropriate, neutrophils were preincubated with Thykamine at 37 °C for 30 min before experiments.

### 2.4. J77A4.1 Cell Culture

J774A.1 monocyte cells (*Mus musculus*) (American Type Culture Collection, Manassas, VA, USA) were grown in Dulbecco’s Modified Eagle Medium (DMEM) 5% serum supplemented with gentamicin 0.05 mg/mL in a moisture-saturated atmosphere containing CO_2_ 5% at 37 °C (VWR Basic CO_2_ Incubator Air jacket 5.3CF). After a first pass, cells were divided (2000 cells/wells) on a 24 well microplates. Cells were pretreated with Thykamine extracts for 24 h in the same culture environment and conditions as growth conditions. After pretreatment, cells were washed twice with DMEM 5% serum and then activated with liposaccharide (LPS, Sigma #L4391-1MG) 100 ng/mL to produce NO for a period of 24 h. NO production was measured in the supernatants using the Griess reagent method and kit (Promega #G2930) and NO concentration were calculated with a standard curve of NO_2_^−^ [11].

### 2.5. Protection of Lipid Peroxidation by Thykamine

Thykamine were tested for its capacity to protect against lipid peroxidation. The assay used was an adaptation of a spectrometric cuvette method [12] to a microplate (Greiner Bio-One UV-Star) test. Lipidic emulsions were prepared with linoleic acid, reactive oxygen species were generated by 2,2-azobis (2-methylpropionamidine) dihydrochloride (AAPH) at 37 °C in continuous stirring at 234 nm for 45 min (Microplate Reader xMark, Biorad). Areas under the curves at 234 nm were calculated and the differences between Thykamine samples.

### 2.6. Detection of SOD in Thykamine

SOD zymography was performed in non-denaturating conditions on a polyacrylamide gel (acrylami-de/bis-acrylamide 30%/10%) [13,14]. Thykamine extracts were added to obtain a final concentration of 0.2 mg proteins. Electrophoresis was run at 80 V for 3 h. Activity was revealed with nitroblue tetrazolium solution (0.1%). Superoxide ions were generated by adding riboflavin. The presence of SOD-like enzyme was revealed by white spots on the blue colored gel.

For the determination of SOD activity, SOD was isolated from Thykamine extract using 0.1 mg/mL in 50 mM potassium phosphate buffer (pH 7.8), 1 mM EDTA, and 2% (*w*/*v*) polyvinylpolypyrrolidone. After centrifugation at 10,000× *g* for 30 min at 4 °C, the supernatant was collected and SOD activity was assayed using the photo-oxidation of riboflavin generating ROS, including O_2_^−^. These anions reduced nitro blue tetrazolium (NBT) at 560 nm [7].

### 2.7. Evaluation of O_2_^−^ Production

The production of O_2_^−^ was determined using the reduction of cytochrome *c*. Neutrophils (10^7^ cells/mL) were preincubated 30 min at 37 °C with diluent or Thykamine and then diluted to 10^6^ cells/mL in the presence of 130 µM cytochrome c, followed by a stimulation with 0.1 µM fMLP for 5 min at 37 °C. Absorbance of supernatants was measured at 540 and 550 nm. Amounts of O_2_^−^ present in supernatants were determined by the difference between the two readings and using an extinction coefficient of 21.1.

### 2.8. Flow Cytometric Analysis of Neutrophils Pretreated with Thykamine

Flow cytometric analyses were performed on neutrophils resuspended in HBSS. Due to the structure of Thykamine, neutrophils pretreated with these thylakoid membranes were directly visible through the spontaneous fluorescence of the compound monitored at an excitation wavelength of 488 nm and an emission wavelength of 675 nm. Samples were analyzed with a flowcytometer EPICS-XL (Beckman Coulter, Miami, FL, USA). FSC was set on linear scales, SSC and fluorescence channels (FL4) were set on logarithmic scales.

### 2.9. Confocal Microscopy of Neutrophils under Thykamine

Neutrophils were preincubated with different concentrations of Thykamine for 30 min before evaluation by confocal microscopy. Neutrophils were resuspended in 50 mL phosphate-buffered saline (PBS), placed on coverslips, air-dryed, and fixed in 4% paraformaldehyde (pH 7.4) for 15 min. They were further washed with PBS before analysis. Images were collected on an Olympus BX-61 confocal laser microscope and analyzed with the application program Fluoview 500.

### 2.10. Monitoring of Neutrophil Chemotaxis by Fluorescence

Chemotaxis of neutrophils was studied by the following two fluorescence-based neutrophil migration methods: Transpolycarbonate migration (TPM) assay and transendothelial migration (TEM) assay [15,16]. TPM assay was performed using a disposable 96-well chemotaxis chamber with an 8 µm pore size polycarbonate filter. Freshly isolated neutrophils (1 × 10^7^ cells/mL) were incubated with 5 µg/calcein-AM for 30 min at 37 °C in the dark. Total fluorescence was obtained from a known number of neutrophils. The test was performed by placing neutrophils on the filter followed by a migration for 1 h (37 °C and 5% CO_2_) in the dark. Non-migrating cells were removed by gently wiping the filter with a tissue. Cell migration was measured with a microplate fluorescence reader (FL600; Bio-Tek Instruments, Winooski, VT, USA) with bottom-read configuration (excitation 485 nm; emission 530 nm) [15].

For TEM assay, human endothelial cells (HUVEC) were seeded out on PVP-free collagen-coated polycarbonate filters (pores of 8 µm) in Transwell culture plate inserts at 1.5 × 10^5^ cells/0.4 mL/culture plate insert. After the obtainment of a complete monolayer of endothelial cells, neutrophils, preincubated with calcein-AM as above, were added to the upper chamber (4 × 10^5^ cells), while the lower chamber was filled with or without 10^−7^ M LTB_4_, IL-8 or fMLP. Transmigration of neutrophils through the endothelial monolayer was allowed to proceed during half an hour. Non-migrating neutrophils were removed and migration of neutrophils was evaluated by the fluorescence measured in the lower chamber with a microplate fluorescence reader (excitation 485 nm; emission 530 nm). In both assays, results were expressed in percentage of cells that migrated toward the chemotactic agent.

### 2.11. Flow Cytometric Analysis of Phagocytosis

Phagocytosis by neutrophils was studied by monitoring the process of internalization of fluorescent labeled bacterial particles as indicated in the Vybrant Phagocytosis assay kit V-6694 (Molecular Probes Inc., Eugene, OR, USA) with slight modifications. Samples were analyzed with a flowcytometer EPICS-XL as above, and the fluorescence intensity was determined at 485 nm excitation and 530 nm emission wavelengths. Results were expressed as arbitrary units of fluorescence of neutrophils that have phagocytized fluorescent particles among 10,000 cells analyzed. 

### 2.12. Analysis of Neutrophil Degranulation

Flow cytometry and ELISA measurements were used to analyze degranulation by neutrophils. Exocytosis of granules was assessing by evaluating the appearance of the granular markers CD63 (primary granules) and CD66b (secondary granules) at the cell surface, using anti-CD63or CD67 antibodies and fluorescent secondary antibody as previously reported [17]. Results were expressed in arbitrary units of fluorescence intensity.

Exocytosis of neutrophils was also studied by assessing the release of MPO and LF in supernatants. Neutrophils (10 million/mL) were stimulated 5 min at 37 °C with vehicle or 10^−7^ M fMLP in the presence or absence of 1 µg/mL cytochalasin B, an actin depolymerizing agent known to amplify granule exocytosis. One hundred (100) µL of supernatant or cells were added to 2.4 mL of a mixture of potassium phosphate buffer, 0.2 mg/mL of O-dianiside dihydrochloride and 500 µL of 0.003% H_2_O_2_. MPO contents were obtained by measuring absorbance at 460 nm and calculated from human MPO calibration. Results were expressed in percentages (ratio extra-cellular/intra- + extra-cellular materials). Release of LF was measured by ELISA [18]. Absorbance was read at 450 nm with a microplate reader, and the LF concentration was calculated by comparison to the human LF calibration curve. Results are expressed in ng/mL.

### 2.13. Analysis of LDH Release

LDH release assay was used to determine the effects of Thykamine on neutrophils viability. Neutrophils and Thykamine were incubated 1 h at 37 °C before centrifugation. Both supernatants and pelleted neutrophils were collected separately for testing. Triton X-100 was used to lyse neutrophils cells. Reaction solution was composed of 1.25 mL substrate (0.14 mg/mL NADH in 0.1 M sodium phosphate buffer, pH 7.35) and 50 μL of pyruvate solution (Sigma, St-Louis, MO, USA) and was added to 50 µL of lysed cell or supernatant samples. Colorimetric readings were measured at 340 nm. Results were expressed as percentages of the ratio of absorbance read in supernatants by the total absorbance (cells plus supernatant).

### 2.14. Statistics

Results are presented as means and ± SEM. Statistical analyses were performed using GraphPad Instat 3.0 (GraphPad Software, Inc., San Diego, CA, USA). Sample groups were analyzed by using paired or unpaired Student’s *t* test. Significance was set at two-tailed *p* value < 0.05.

## 3. Results

### 3.1. Effects of Thykamine in Inflammatory Animal Models

**Figure 1:**
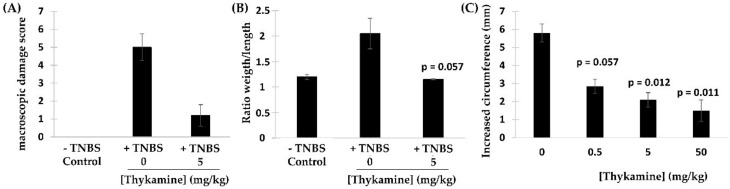
In vivo effects of Thykamine in animal models of acute inflammation. Hapten 2,4,6-trinitrobenzenesulfonic acid (TNBS) induced colitis in rats was measured by (**A**) the macrocsopic damage and (**B**) the ratio weight/length. Male Wistar rats, after an overnight food deprivation, were anesthetized with isoflurane before insertion of a colonic catheter of 8 cm. The TNBS (25 mg/mL in 50% aqueous/ethanol; vol/vol) was injected into rat colon (total volume injected: 1 mL/rat). The control rats (*n* = 4) received 1 mL of vehicle (aqueous/ethanol; vol/vol) intracolonically. Rats were injected intraperitoneally with Thykamine (5 mg/kg, *n* = 4) in sterile physiologic saline (1 mL) immediately prior to anesthesia. Statistics: Paired 2-tailed *t* test (significance for *p* value < 0.05). (**C**) Carrageenan-induced paw edema in rats. Male Wistar rats pre-fasted overnight received Thykamine (0, 0.5, 5 or 50 mg/kg; *n* = 9, 5, 6, 5, respectively) in sterile physiologic saline by intraperitoneal injection (1 mL) immediately prior to subplantar injection of carrageenan (0.1 mL of 1% suspension in 0.9% saline) in the right hind paw. Paw circumference was measured immediately prior to carrageenan injection and 5 h afterwards. Edema was expressed in mm as the increase of paw circumference measured after carrageenan injection and compared to the pre-injection value of individual animals. Statistics: paired 2-tailed *t* test (significance for *p* value < 0.05).

To study the possible in vivo anti-inflammatory effects of the natural thylakoid extract (hereby refers to as Thykamine) in animals, model of acute inflammation targeting two different tissues including the colon wall and the paw structure were investigated (Figure 1). Inflammation of the colon wall tissues was induced in presence of the hapten 2,4,6-trinitrobenzenesulfonic acid (TNBS) and was evaluated using two parameters: The macroscopic damage score and the ratio weight/length [19]. The macroscopic damage of tissues from the TNBS-induced lesion was recorded at 5.0 ± 0.7 (*n* = 4), a score corresponding to major ulcerative sites of damage extending more than 1 cm along the length of colon (Figure 1A). The dosage for the animal study was based on a US-patent from M. Purcell. In this patent, they used a dosage of 25 mg/kg in Wistar rats. The average male weight being 200 g we designed a dose response of Thykamine which included 25 mg/kg (5 mg) and 10 time lower (0.5 or higher 50).

Thykamine pretreatment of rats (5 mg/kg intra-peritoneal (i.p.)) significantly reduced this inflammatory score to 1.3 ± 0.6 (corresponding to localized hyperemia with no ulcers) (Figure 1A). In similar experimental conditions, the ratio weight/length of altered tissues was also reduced from 0.200 ± 0.044 (rats without Thykamine) to 0.112 ± 0.002 (rats with Thykamine) (Figure 1B). Altogether, these results indicate that treatment with Thykamine in rats reduce the colonic damage subsequently induced by hapten in vivo.

The effect of Thykamine was also investigated within the in vivo rat model of acute inflammation, i.e., the carrageenan-induced hindpaw edema [20]. To evaluate the edema induced by intraplantar injection of carrageenan, paw thickness was evaluated by measuring the circumference of the paw (expressed in mm), immediately prior to carrageenan injection and after 5 h. Carrageenan-induced edema of rat hindpaw displayed a circumference of 5.6 ± 0.9 mm (*n* = 9) (Figure 1C). Rats treated with i.p. injection of Thykamine were shown to have significantly lower hindpaw circumferences compared to untreated rats (Figure 1C). Indeed, Thykamine at 5 mg/kg significantly reduced the edema by 60% (hindpaw circumference, 2.1 ± 0.4 mm; *n* = 6). These results strongly suggest that treatment with Thykamine in rats has the capacity to diminish the acute inflammation subsequently induced by the red seaweed carrageenan in vivo.

### 3.2. Protection of Thykamine against Oxidative and Nitrosative Stress

**Figure 2:**
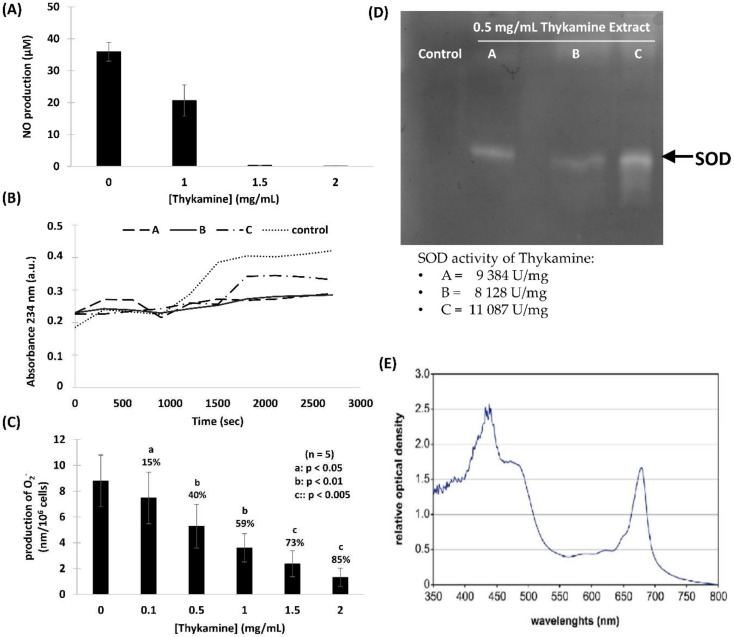
In vivo effects of Thykamine on oxidative stress and protective antioxidant components of Thykamine. (**A**) Protection of Thykamine against NO generated by stimulated J774A.1 cells. J774A.1 cells were pretreated with Thykamine extracts. After removing of Thykamine, LPS 100 ng/mL was added to produce NO for a period of 24 h. NO production was measured in the supernatants using the Griess reagent method and kit (promega #G2930). Results are expressed as mean +/− SEM (*n* = 5). (**B**) Protection Thykamine against lipid peroxidation. Thykamine at 0.5 mg/mL were incubated for 45 min with linoleic acid 0.15 mM at 37 °C in microplates. The reaction was started with AAPH 0.7 mM. Each curve represents either control or different 0.5 mg/mL Thykamine extracts (**A**–**C**). Fresh controls were prepared to test each Thykamine extracts and were combined togheter to present as control on the figure. (**C**) Protection of Thykamine against the production of O_2_^−^ by normal human blood neutrophils stimulated by fMLP. Neutrophils pretreated 30 min with graded concentrations of Thykamine (or HBSS as negative control) were incubated 5 min at 37 °C in the presence of 0.1 µM fMLP. Superoxide anions were evaluated by the reduction of cytochrome *c*. The optical density of the supernatants was read at 540 and 550 nm. Results are expressed in mean ± SEM of nM O_2_^−^/10^6^ neutrophils (*n* = 5). Statistics were performed using Student’s unpaired *t* test (comparison between Thykamine-treated neutrophils versus control neutrophils in HBSS). Abbreviations are a is *p* < 0.05; b is *p* < 0.01 and c is *p* < 0.005. (**D**) SDS-PAGE gel electrophoresis of three 0.5 mg/mL Thykamine extracts (**A**–**C**) and zymography to show corresponding SOD activity. (**E**) Spectroscopic characterization of antioxidant pigments of Thykamine. Light spectroscopy was performed on active Thykamine that showed two major peaks. The first one recorded between 430 and 440 nm was chlorophylls with a shoulder between 460 and 490 nm corresponding to carotenoids. The second peak at 680 nm was chlorophylls (*a* more than *b*). This profile is representative of four different extract of Thykamine.

The two rat models of acute inflammation used above have demonstrated the anti-inflammatory potential of Thykamine. The impact of this natural product on oxidative and nitrosative stress was studied using cells and in vitro models. First, the production of NO by inducible NO synthase (iNOS) was assayed (Figure 2A). It is known that inducible iNOS can generate large amounts of NO in response to agent such as lipopolysaccharide (LPS) and is involved in inflammatory pathologies [21]. The ability of Thykamine extract to inhibit NO production is presented in Figure 2A. Stimulated J774A.1 cells with LPS generated 35 μM of NO. Pre-treatment of these cells 24 h with 1.5 and 2 mg/mL of Thykamine totally prevented the generation of NO whereas a 50% reduction was observed in J774A.1 cells pre-treated with 0.5 mg/mL of Thykamine (Figure 2A). These results suggest that Thykamine is involved in the protection against nitrosative stress producing deleterious NO derivatives. Lipid peroxidation is considered as a general consequence of oxidative stress originating from reactive oxygen species (ROS) altering biological membranes. An emulsion of linoleic acid was prepared to determine the protective effect of Thykamine (0.5 mg/mL) on lipidic stress. It is shown on Figure 2B that peroxidation of linoleic acid was inhibited in presence of different 0.5 mg/mL Thykamine extracts (A–C), suggesting that proliferation of free radicals was diminished. Linoleic acid not treated with Thykamine undergoes a regular increase in peroxidation corresponding to the production of conjugated dienes, absorbance levels at 234 nm reaching 0.42 absorbance units (Figure 2B). Thykamine extracts A and B did not exceed 0.25 optical density at 234 nm while extract C reached 0.35 absorbance units. In neutrophils, it is well-known that ROS have microbicidal and antifungal roles. However, excessive ROS release is also responsible for tissue damage associated with inflammation [22]. Neutrophils preincubated with graded concentrations of Thykamine dose-dependently decreased their production of O_2_^−^ response to 0.1 µM N-formyl-methionyl-leucyl-phenylalanine (fMLP) (Figure 2C). The anion production by fMLP-activated neutrophils was significantly reduced from 15% to 85% by concentrations of Thykamine ranging from 0.1 to 2.0 mg/mL, respectively.

The protective effects of Thykamine in oxidative and nitrosative stress may be explained by the high concentrations of antioxidant enzymes of molecules found in these extracts. Active enzymes such as SOD are known to participate in the reduction of ROS contributing to the reduction of inflammation. SOD activity can be rapidly visualized using zymography [13,14]. SOD activity was measured in Thykamine extracts with white bands observed on the SDS-PAGE gel obtained by a decreased in O_2_^−^ formed by the reaction between nitro blue and riboflavin (Figure 2D). SOD activity reached 1850 U/mg of total proteins demonstrating that Thykamine contains active enzymes able to protect against O_2_^−^ generated by oxidative stress. Thykamine also contains antioxidant molecules that were spectroscopically characterized by two major peaks (Figure 2E). The first one recorded between 430 and 440 nm was related to chlorophylls (predominance of chlorophyll b) with a shoulder between 460 and 490 nm corresponding to carotenoids. The second peak recorded at 680 nm was sharp and related to chlorophylls with predominance of chlorophyll a (Figure 2E). The results suggest the presence of carotenoids and xanthophylls (lutein, neoxanthin, violaxanthin) in Thykamine extracts. Both enzymes (e.g., SOD) and molecules (e.g., chlorophylls, carothenoids) in Thykamine may act in concert to prevent and reduce oxidative stress known to cause inflammation.

### 3.3. Effects of Thykamine on Neutrophils

**Figure 3:**
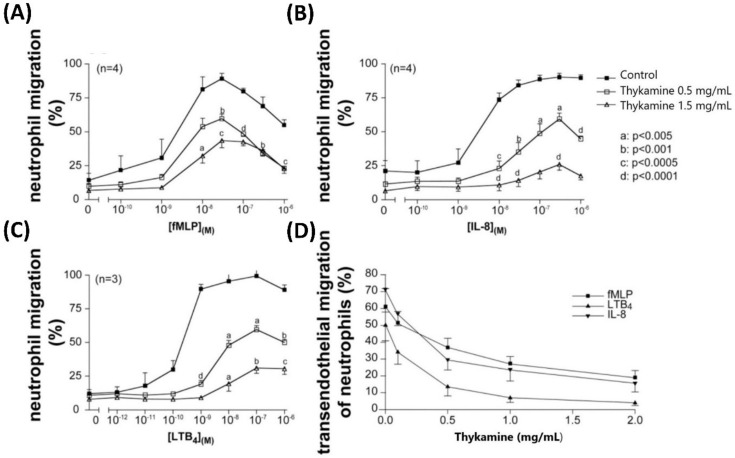
In vitro effects of Thykamine on chemotaxis of normal human blood neutrophils in the presence of fMLP, IL-8 or LTB4. Neutrophils charged with calcein-AM were treated with Thykamine (or HBSS as control) for 30 min before their induced-migration across polycarbonate filters using concentration gradient (10^−12^–10^−6^ M) of fMLP (**A**), IL-8 (**B**) and LTB4 (**C**), and across a monolayer of endothelial cells using 10^−7^ M fMLP, IL-8 and LTB4 (**D**). Results are expressed in percentages (mean ± SEM of triplicates) of fluorescent cells that migrated through the filters or the endothelial monolayers with respect to total number of cells loaded. Statistics were performed using Student’s unpaired *t* test (comparison between Thykamine-treated neutrophils versus control untreated neutrophils in HBSS). Statistics were performed using Student’s unpaired *t* test (comparison between Thykamine-treated versus control cells in HBSS) where significance for *p* value is a: *p* < 0.005; b: *p* < 0.001; c: *p* < 0.0005 and d: *p* < 0.0001.

Since the two rat models of acute inflammation used above to demonstrate the anti-inflammatory potential of the natural product Thykamine are critically dependent on neutrophils [20,23], the next step was to study if Thykamine could impact directly the activity of human blood neutrophils in vitro (Figure 3). During the inflammatory process, the first cells to invade pathologic tissues are neutrophils that rapidly respond to chemotactic agents. By reducing neutrophil chemotaxis, a compound can reduce the inflammatory process related to the local presence of activated neutrophils in excess. To avoid non-specific effects of Thykamine on polycarbonate membrane or on HUVECs used to study neutrophil chemotaxis, neutrophils preincubated with Thykamine were washed twice to remove unfixed compounds. As a prerequisite, we initially evaluated the effect of Thykamine on neutrophils viability by measuring the release of LDH. Thykamine at concentrations up to 5 mg/mL (37 °C, 1 h) had no effect on lactate deshydrogenase (LDH) release by human blood neutrophils.

Freshly isolated neutrophils responded to a gradient of chemotactic factors by agonist-directed movement (non-random migration) toward these factors. Neutrophil chemotaxis through a polycarbonate membrane rapidly increased with augmentation of concentrations of chemotactic agents: fMLP and leukotriene B_4_ (LTB_4_) were associated with biphasic curve of chemotaxis (Figure 3A,C), IL-8 with a plateau (Figure 3B). Neutrophil chemotaxis was significantly induced by fMLP and IL-8 with a similar threshold of 10^−8^ M, and by LTB4 with a threshold of 10^−9^ M. Thykamine dose-dependently reduced neutrophil chemotaxis toward the chemotactic factors studied (Figure 3). However, patterns of chemotaxis inhibition by Thykamine differ depending on the chemotactic agent. The most efficient effect of Thykamine at 1.5 mg/mL was recorded with neutrophils incubated in the IL-8 gradient, reducing their chemotaxis by 100% at 10^−8^ M IL-8, and by 78% at 10^−6^ M IL-8 (Figure 3B). Thykamine at 0.5 mg/mL decreased neutrophil chemotaxis by 61% at 10^−8^ M IL-8, and by 56% at 10^−6^ M IL-8. Similarly, 1.5 mg/mL Thykamine reduced neutrophil chemotaxis by 100% in the presence of 10^−9^ M LTB4 and by 67% at 10^−6^ M LTB4. Also, Thykamine at 0.5 mg/mL decreased neutrophil chemotaxis by 81% at 10^−9^ M LTB4, and by 50% at 10^−6^ M LTB4 (Figure 3C). Interestingly, the chemotactic response of Thykamine-pretreated neutrophils to fMLP was different from the one to IL-8 and LTB4. Thykamine at 0.5 and 1.5 mg/mL reduced neutrophil chemotaxis by 36 and 53% at 10^−8^ M fMLP, respectively. When fMLP concentrations increased from 10^−7^ M to 10^−6^ M, the inhibitory effects of Thykamine at 0.5 and 1.5 mg/mL on neutrophil chemotaxis were equivalent (Figure 3). At 10^−6^ M fMLP, 0.5 and 1.5 mg/mL Thykamine inhibited chemotaxis by 39%. To further validate the inhibitory effect of Thykamine on neutrophil chemotaxis, studies were performed with a monolayer of normal human endothelial cells. As shown in Figure 3D, Thykamine dose-dependently inhibited the induced migration of normal human blood neutrophils. In the presence of chemotactic agents at 10^−7^ M, the best inhibitory effect of Thykamine was recorded with LTB4 that reached 95% inhibition of the control LTB4-induced chemotaxis. Altogether, the results suggest that Thykamine treatments reduce chemotaxis of neutrophils, which may participate to reducing inflammation.

### 3.4. Effects of Thykamine on Phagocytosis by Neutrophils

**Figure 4:**
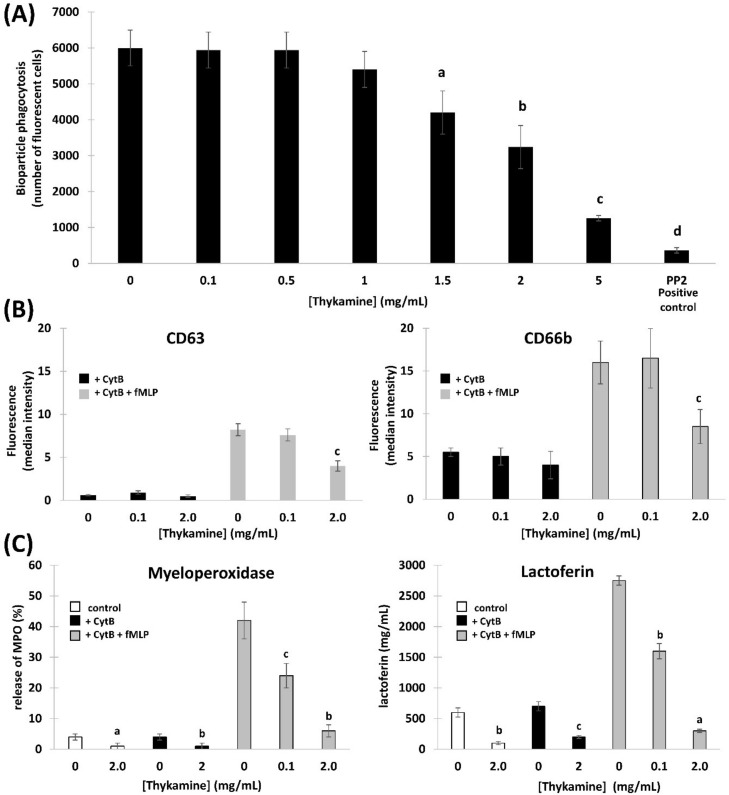
In vivo effect of Thykamine on phagocytosis (**A**) and on granule functions (**B**,**C**) in normal human blood neutrophils. (**A**) Phagocytosis of fluorescein-labeled *Escherichia coli* BioParticles by normal human blood neutrophils. Neutrophils pretreated 30 min with graded concentrations of Thykamine (or HBSS as negative control, and 10 µM phosphoprotein phosphatase PP2 as positive control) were incubated 30 min at 37 °C in the presence of homogenized FITC-labeled *E. coli* BioParticles. Trypan blue was added to quench the extracellular fluorescence of non-phagocytosed *E. coli* BioParticles before analysis by cytofluorometry (485 nm excitation, 530 nm emission wavelengths). Results are expressed in number of fluorescent neutrophils that have phagocytized fluorescent particles among 10,000 cells analyzed. Statistics were performed using Student’s unpaired *t* test (comparison between Thykamine-treated neutrophils versus control neutrophils in HBSS) of *n* = 7 where significance for *p* value is a: *p* < 0.001; b: *p* < 0.005; c: *p* < 0.0001 and d: *p* < 0.0005. (**B**,**C**) Release of primary and secondary granules by normal human blood neutrophils.Neutrophils were pretreated 30 min with 0.1 or 2 mg/mL Thykamine (or HBSS as negative control) before stimulation with vehicle or 10^−7^ M fMLP for 5 min at 37 °C in the presence or absence of 1 µg/mL cytochalasin B (CytB) with or without fMLP. Statistics were performed using Student’s unpaired *t* test (comparison between Thykamine-treated versus control cells in HBSS) where significance for *p* value is a: *p* < 0.001; b: *p* < 0.005 and c: *p* < 0.01. (**B**) Degranulation was monitored by the appearance of CD63 (primary granules) and CD66b (secondary granules) at the cell surface, as analyzed by cytofluorometry. Results are expressed in arbitrary units of fluorescence intensity (mean ± SEM; *n* = 3). (**C**) Degranulation was assessed by the release in supernatants of MPO and LF. MPO was evaluated by comparison to a calibration curve created with known dilutions of human MPO. LF was measured by ELISA using a peroxidase-conjugated anti-rabbit Ab. Results of MPO and LF are expressed in percentages (ratio extra-cellular/intra- + extra-cellular materials) and in ng/mL, respectively.

Once the neutrophil has transmigrated to an inflammatory site, its function is primarily adherence to, and phagocytosis of the, foreign material, function that further leads to activate various signaling and metabolic pathways [24]. Thykamine, at lower concentrations up to 1.0 mg/mL, did not modify neutrophil phagocytosis of bioparticles (Figure 4A). However, at higher concentrations (e.g., 1.5 to 5 mg/mL), a significant dose-dependent reduction of the capacity of neutrophils to ingest particles was observed (Figure 4A). For instance, neutrophils preincubated with 2 mg/mL of Thykamine phagocytized 46% less particles compared to control untreated cells (Figure 4A). Interestingly, Thykamine was able to greatly decrease neutrophil phagocytosis (79% inhibition) at concentration as high as 5 mg/mL. 

Neutrophils have also the capacity to kill microbes via a ROS independent pathway by the release of granule-stored proteins into the phagolysosome or in the extracellular medium [22]. However, such neutrophil degranulation can also cause inflammation by the extensive and sustained release of proteolytic enzymes. The reduction of such excessive release of granule enzymes is often associated with reduced inflammation. We first tested the effects of Thykamine on neutrophil degranulation using flow cytometry of the membrane translocation of two fluorescent granule markers, CD63 and CD66b (primary and secondary granules, respectively; Figure 4B). Thykamine, at concentrations of 2.0 mg/mL, was able to decrease the appearance of fluorescent markers at the neutrophil membrane by 50% for both primary (CD63) and secondary (CD66b) granules (Figure 4B). To further confirm these data, degranulation was studied by directly measuring the release in supernatants of proteins such as myeloperoxidase (MPO; primary granules) and lactoferrin (LF; secondary granules) by fMLP-activated neutrophils (Figure 4C). Thykamine, at concentrations of 0.1 and 2.0 mg/mL, significantly inhibited the release of MPO by 43% and 86%, respectively. Interestingly, 2.0 mg/mL Thykamine were able to abrogate the spontaneous degranulation of MPO (Figure 4C). Similarly, Thykamine, at concentrations of 0.1 and 2.0 mg/mL, significantly inhibited the release of LF by 46% and 90%, respectively, and at 2.0 mg/mL Thykamine strongly reduced the basal output of LF release (Figure 4C).

### 3.5. Morphological Characterization of Neutrophils Incubated with Thykamine

**Figure 5:**
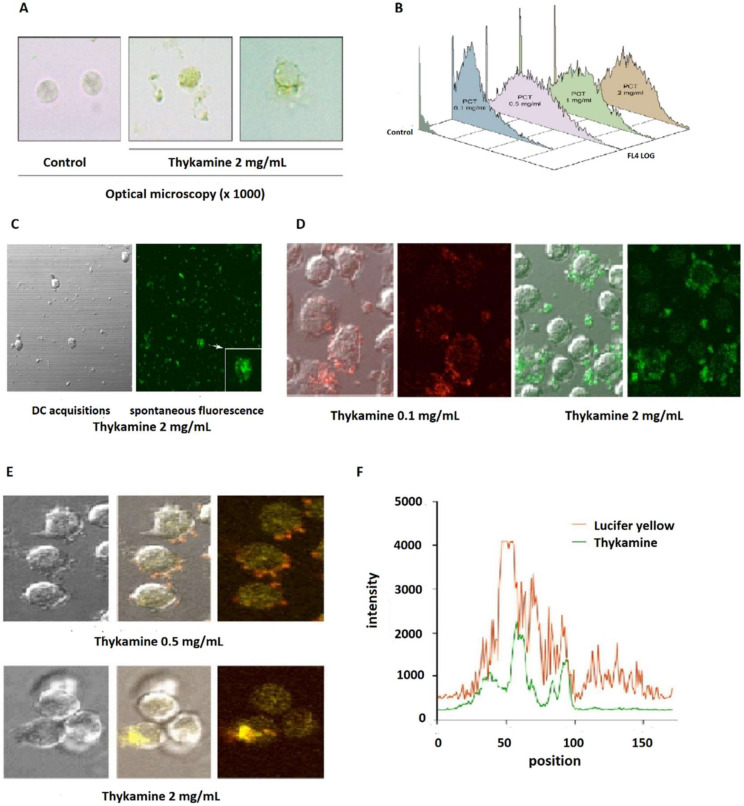
Morphological characterization of normal human blood neutrophils in the presence of Thykamine at 1000× magnification times. (**A**,**B**) granularity measured and visualized by epifluorescence microscopy (**A**) and by flow cytometry (**B**) with spontaneous fluorescence of Thykamine. Neutrophils were preincubated with graded concentrations of Thykamine at 37 °C for 30 min. FSC was set on linear scales, SSC and fluorescence channels (FL4) were set on logarithmic scales. (**C**,**D**) Confocal microscopy of normal human blood neutrophils pre-treated with Thykamine. Neutrophils were preincubated with different concentrations of Thykamine for 30 min before evaluation by confocal microscopy (Olympus BX-61 confocal laser microscope). (**C**) the analysis of neutrophils pre-treated with 2 mg/mL Thykamine was performed by using differential interference contrast (DIC) and three-dimensional confocal slices (0.2 µm); the arrow indicates a medial view of a Thykamine-treated neutrophil. (**D**) Confocal microscopy visualization of human blood neutrophils pre-treated with two different concentrations of Thykamine. (**E**,**F**) Characterization of Thykamine entry into human blood neutrophils by the uptake of Lucifer Yellow. Neutrophils were incubated in the simultaneous presence of 0.5 or 2 mg/mL Thykamine and Lucifer Yellow (LY) and Thykamine penetration proceeded for 10 min at 37 °C. (**E**) visualization of Thykamine and LY was performed with confocal microscopy (Olympus BX-61 confocal laser microscope). (**F**) computerized comparison of the respective position of Thykamine particles and Lucifer Yellow while penetrating neutrophils. (PCT = Thykamine extract in this figure).

The spontaneous fluorescence of Thykamine (emission at 488 nm, extinction at 675 nm) permitted analysis of neutrophils treated with increasing concentrations of Thykamine by laser spectrophotometry and confocal microscopy. It is also useful to point out that Thykamine suspensions are green with the possibility of visualizing it by conventional optical microscopy. These characteristics give invaluable tools to follow the Thykamine lipido-protidic complex throughout the cells. Thus, neutrophils preincubated with Thykamine, at 37 °C for 30 min, were morphologically unaltered and Thykamine gave an apparent partial green wrapping of these neutrophils, as visualized by optic microscopy (Figure 5A). It is also noteworthy that Thykamine wrapping neutrophils (or that has been incorporated by the cells) remained spontaneously fluorescent at 675 nm. Surprisingly, flow cytometry analysis of Thykamine-treated neutrophils showed modifications mainly of their forward angle light scatter. As shown in the Figure 5B, neutrophils preincubated with increasing concentrations of Thykamine were shown to have an increased granularity (forward scatter (FSC)) that remained similar from 0.5 to 2 mg/mL of Thykamine. Freshly isolated neutrophils (control) were not fluorescent and their light scatters (FSC and SSC (side scatter)) were low and equivalent to the background noise (Figure 5B).

The increase of neutrophil granularity following pretreatment with Thykamine was also visualized by confocal microscopy (Figure 5C,D). Differential interference contrast acquisitions showed that Thykamine particles had different forms from very small to large particles (maximum 4–5 mm) located in contact of neutrophils and in the extracellular media (Figure 5C). All Thykamine particles were fluorescent and easily identifiable. Thus, the enlargement of a Thykamine-treated neutrophil (medial view) showed Thykamine particles irregularly concentrated inside the cell. At concentration of 0.1 mg/mL, the majority of Thykamine particles were essentially detected within the neutrophils, and very few particles were detected extracellularly (Figure 5D). Thus, Thykamine particles seem to be attracted toward and enter the neutrophil easily and rapidly. To decipher mechanisms of Thykamine penetration into neutrophils, cells were incubated with Thykamine and Lucifer Yellow, a useful probe for fluid-phase pinocytosis [25]. Thykamine particles and the dye added simultaneously to cells were rapidly visualized inside the neutrophils (Figure 5E). The confocal microscopic analysis of cell penetration of the two compounds was computerized by studying an arbitrary straight line drawn through the cell (Figure 5F). The comparison of the respective position of Thykamine particles and Lucifer Yellow while penetrating the cell allowed us to show a strong similarity between both compounds. This result indicates that Thykamine particles entered the neutrophils, at least in part, by pinocytosis.

## 4. Discussion

Results presented in this article demonstrated the high properties of Thykamine extracts to regulate and inhibit inflammation through their modulating activity on physical and biochemical parameters of inflammation. Thykamine injected intraperitoneally to rats reduced the inflammatory process of TNBS-induced colitis and carrageenan-induced paw edema (Figure 1). Thykamine extracts also protected against lipid peroxidation and inhibit NO production in J774A.1 cells (Figure 2). The presence of SOD enzyme having a relevant superoxide inhibition activity as well as carotenoids in Thykamine extracts could account for the prevention against lipid peroxidation (Figure 2). Moreover, SOD inhibits the formation of ONOO^−^ by decreasing superoxide levels that prevent the peroxidant effects produced by ONOO^−^. Asada (2006) has demonstrated that thylakoid membranes, forming Thykamine extracts, contained Cu/Zn-SOD enzymes [26]. Glycolipids composition of thylakoid membranes of Thykamine could also be involved in the inhibition of NO production since a treatment with these lipids blocked LPS-induced Inducible nitrite oxide synthase (iNOS) expression in HUVECs [27]. The bilayer lipid membranes of Thykamine extracts are constituted of glycolipids including monogalactosyl diacylglycerol (MGDG), digalactosyl diacylglycerol (DGDG) and sulfoquinovosyl diacylglycerol (SQDG) [27].

The involvement and role of redox molecules, such as NO and ROS, as key mediators of immunity have recently gained improved attention and appreciation. Physiological mechanisms conducting to an imbalance in these species are known to be implicated in diseases such as diabetes, cancer, heart and lung disease, autoimmune diseases, ageing and various infectious diseases. Beside SOD and other antioxidants found on Thykamine membranes, a possible mechanism of action of this anti-inflammatory botanical agent could be the binding between [2Fe-2S] clusters, located on Thykamine, and the cysteine of the enzyme glutaredoxin (Grx1). It is now reported that Grx1 accumulated in various inflammatory disease [28] having a pro-inflammatory activity [29]. Thus, development of Grx1 inhibitors could be efficient agents for anti-inflammatory activity. The binding of Grx1 on Thykamine could adduct the nucleophilic active site of Grx1 and explain its anti-inflammatory activity.

Results presented in this study show that Thykamine extract isolated from spinach have antioxidant properties that could explained their ability to modulate phlogogenic functions of human blood neutrophils. Moreover, the resolution of inflammation in two rat models could be explained by the effects of Thykamine extracts on human neutrophils.

Primary functions of neutrophils, like ROS production and degranulation following chemotaxis toward and phagocytosis of foreign particles abnormally present in tissues, are associated with an obligatory inflammatory response. However, this inflammatory reaction can pathologically persist with deleterious impact on tissues and subject health. Normal human blood neutrophils were shown to be modulated dose-dependently when preincubated with Thykamine that decreased the production of O_2_^−^, and degranulation of primary and secondary granules stimulated by fMLP, as well as chemotaxis in response to fMLP, IL-8 or LTB4, and phagocytosis of bioparticles. Note that 2 mg/mL Thykamine inhibited 85% of fMLP-induced production of O_2_^−^ (Figure 2C). As the Thykamine extracts were found to contain relevant SOD activities and carotenoid concentration, Thykamine could interfere with neutrophil functions through these natural antioxidants. SOD activities scavenging increase glutathione enzymes as well as carotenoids from spinach and have an antioxidant activity on human erythrocytes by increasing gluthatione reductase and decreasing catalase, an effect mainly related to lutein [30]. Interestingly, β-carotene has been reported to abrogate the generation of ROS induced by *para*-nonylphenol in human blood neutrophils and in whole blood in vitro [31]. These data suggest that Thykamine extracts, at least in part through SOD and carotenoids, could impact on neutrophil machinery by modulating signals associated with the regulation of NADPH-oxidase. It is also useful to stress that 0.1 to 2 mg/mL Thykamine linearly inhibited (from 15 to 85%) neutrophil production of O_2_^−^ (Figure 2C). These data suggest that carotenoids in the thylakoid complex Thykamine did not show any pro-oxidant actions as reported with high concentrations of pure carotenoids [4,32]. Similarly, carotenoids can have, depending on concentrations, anti- or pro-apoptotic effects on tumor cells [33]. Beta-carotene cleavage products have been recently reported to have effects on spontaneous apoptosis of human neutrophils [34]. Results on the absence of effects of Thykamine on neutrophil viability suggest that carotenoids contained in Thykamine do not impact neutrophil apoptosis. Moreover, our results indicate that β-carotene in Thykamine was not associated with pro-apoptotic cleavage products, or if so Thykamine was capable of their neutralization. This is particularly important since β-carotene elevated concentrations and cleavage products have been related to neutrophil toxic effects, especially in smokers [35,36].

Considering the other primary functions of human blood neutrophils that are involved in inflammation, we showed significant inhibition by Thykamine extracts of several processes including; (1) chemotaxis of human blood neutrophil induced by three different agonists (Figure 3), (2) phagocytosis of bioparticles by neutrophils, and (3) degranulation of neutrophil via the release of primary and secondary granules induced by fMLP (Figure 4). Interestingly, β-carotene intake alone was shown to have no effect or increasing effect on neutrophil chemotaxis in cows suggesting that this anti-oxidant molecule alone was not related to the inhibitory effects of Thykamine extracts on human neutrophil chemotaxis [37,38]. It is also noteworthy to stress that Thykamine extracts were able to down-regulate the spontaneous degranulation of human blood neutrophils in vitro, an effect that could be associated with a reduction of their spontaneous apoptosis. Finally, the inhibitory effect of Thykamine on neutrophil phagocytosis, that was only partial (46% inhibition at 2 mg/mL Thykamine (Figure 4), could be a beneficial anti-inflammatory effect while this major neutrophil function of phagocytosis was maintained to a certain extent. This reduction of neutrophil phagocytosis by Thykamine could be explained by SOD enzyme since it has been shown that exogenously added SOD induced neutrophil apoptosis by the dismutation of superoxide radicals [39]. It is noteworthy that, for the first time, a natural product has been shown to reduce phagocytosis by normal human neutrophils.

Different techniques of microscopy allowed us to localize easily Thykamine particles, that are spontaneously fluorescent, in the extra-cellular milieu, as well as Thykamine particles wrapping and inside the cells with dose-dependent modifications of granularity (Figure 5A). Short-term incubations (<1 h) that showed various inhibitory effects of Thykamine on neutrophil functions indicate a rapid cellular penetration of this complex. A major function of defense supported by neutrophils is rapid phagocytosis of foreign particles [40]. This could be the case for Thykamine particles. However, the data presented in Figure 5E,F using Lucifer Yellow dye indicate that Thykamine particles also entered the cell by pinocytosis. This mode of cell entry could be supported by a report on the effects of carotenoids on gap junctions. Carotenoids were showed to significantly upregulate expression of connexin 43 protein and gap junctional intercellular communication, as demonstrated by Lucifer Yellow dye transfer [41]. Thus, it is possible that Thykamine, through their carotenoids, is capable of a rapid entry in neutrophils by pinocytosis. This rapid penetration of Thykamine in neutrophils could be also associated with effects on the complex neutrophil enzyme machinery involved in the inflammatory process.

Further investigations are needed to decipher the exact mechanisms of anti-inflammatory action of this complex natural product that may require the integrity of its lipido-protidic composition to remain active. It is now recognized that phosphoinositide 3-kinases (PI3Ks) are important signaling lipid kinases that regulate multiple biological functions [42]. For example, increased PI3K activity has been observed in tumor cells [43]. Different isoform of PI3K plays specific roles in different cell types. The PI3K isoform p110∂, mainly expressed in white blood cells, plays a key role in the immune system. For example, p110δ PI3K plays a main role in promoting neointimal thickening and inflammatory processes during vascular stenosis, with its inhibition providing significant reduction in restenosis following carotid injury [44]. The p110δ also is involved in B-cell antigen and IL-4 receptor signaling [45]. Since PI3K isoform p110∂ has a key role in immune system, it would be interested to verify how it is influenced or inhibited by Thykamine.

In conclusion, the present report adds a new dimension to the anti-inflammatory effect of the natural product Thykamine from spinach thylakoids previously suspected in human macrophages [6]. Thykamine has in vivo anti-inflammatory effects in two animal models of acute inflammation directly related to the presence of neutrophils. As a corollary, Thykamine extracts significantly impact human neutrophil primary functions by reducing their possible toxic effects during the inflammatory process.

## Abbreviations

AAPH	2,2-azobis (2-methylpropionamidine) dihydrochloride
CD63	Primary granule membrane molecules
CD66b	Secondary granule membrane molecules
DGDG	Digalactosyl diacylglycerol
DMEM	Dulbecco’s Modified Eagle Medium
FITC	Fluorescein isothiocyanate
fMLP	N-formyl-methionyl-leucyl-phenylalanine
FSC	Forward scatter
Grx	Glutaredoxin
HBSS	Hank’s Balanced Salt solution
LDH	Lactate deshydrogenase
LF	Lactoferrin
IL	Interleukin
iNOS	Inducible nitrite oxide synthase
i.p.	intra-peritoneal
LPS	Lipopolysaccharide
LTB_4_	Leukotriene B4
MGDG	Monogalactosyl diacylglycerol
MPO	Myeloperoxidase
NO	Nitric oxide
O_2_^−^	Superoxide anion
PBS	phosphate buffered saline
PSI	Photosystem I
PSII	Photosystem II
RNS	Reactive nitrogen species
SOD	Superoxide dismutase
SSC	Side scatter
SQDG	Sulfoquinovosyl diacylglycerol
TEM	transendothelial migration
TNBS	2,4,6-trinitrobenzenesulfonic acid
TPM	transpolycarbonate migration
